# Supplementary material for: Birth Weight and Early Postnatal Outcomes: Association with the Cord Blood Lipidome
Source: Nutrients. 2022 Sep 12;14(18):3760. doi: 10.3390/nu14183760 (PMC9505183; doi:10.3390/nu14183760)

Figure S1. (**A, B**) PCA, (**C, D**) PLS-DA, and (**E-J**) OPLS-DA score plots (green circles, SGA samples (n = 12); purple squares, AGA samples (control group, n = 12); blue triangle, LGA samples (n = 12); orange diamonds, QC samples (n = 8)) of LC-MS positive and negative ion modes. **C** (R^2 = 0.921; Q^2 = 0.784, p CV-ANOVA = 2.01x10^{-12}), and **D** (R^2 = 0.989; Q^2 = 0.972, p CV-ANOVA = 1.36x10^{-28}) represent the PLS-DA models built for LC-MS ESI (+), and LC-MS ESI (−), respectively.

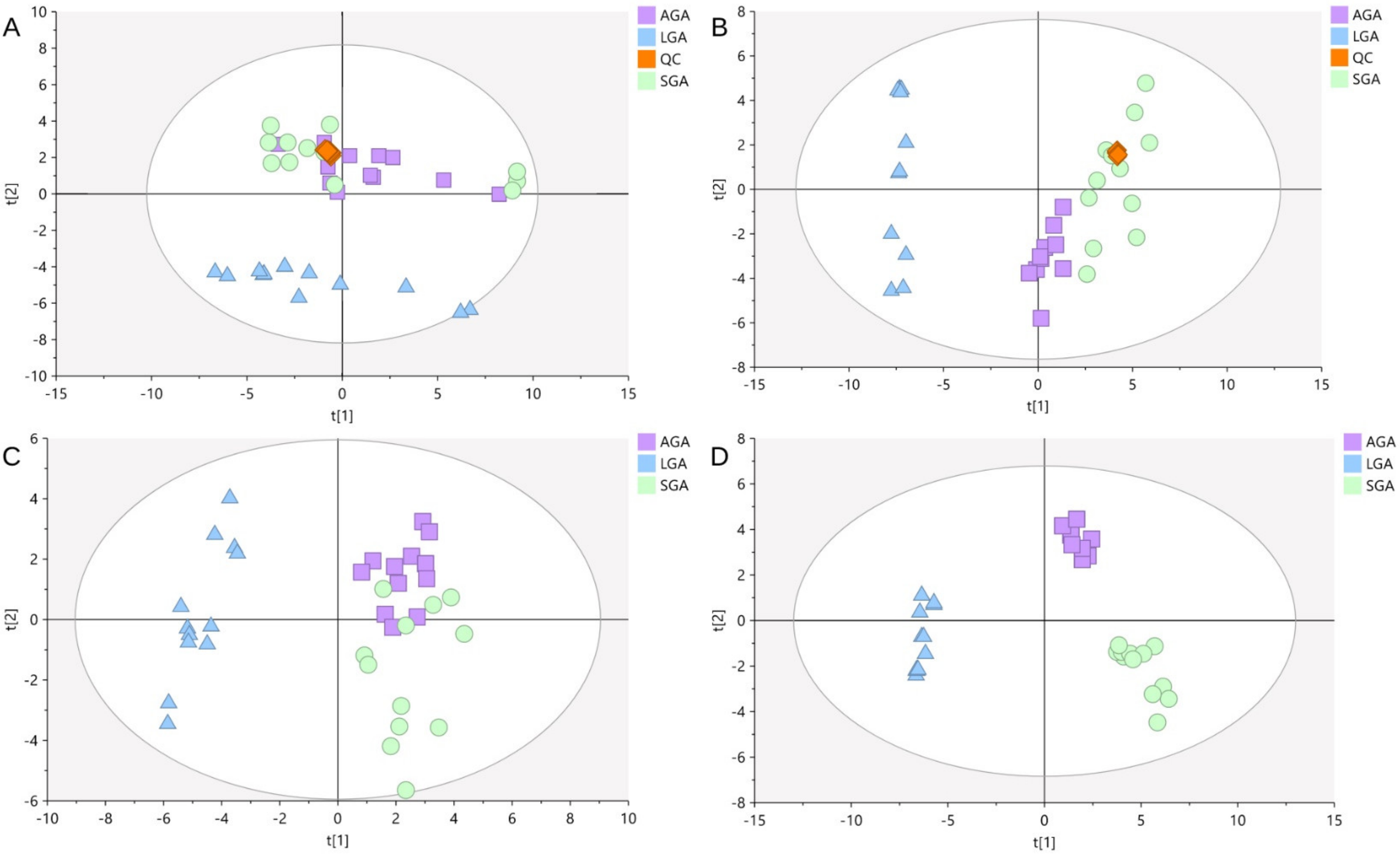

Supplement: Supplementary file 1 [file nutrients-14-03760-s001.zip › Supplementary Figure S1.pdf]
