# Supplementary material for: Birth Weight and Early Postnatal Outcomes: Association with the Cord Blood Lipidome
Source: Nutrients. 2022 Sep 12;14(18):3760. doi: 10.3390/nu14183760 (PMC9505183; doi:10.3390/nu14183760)

Figure S2. A SGA vs AGA (R^2 = 0.989; Q^2 = 0.619, 92% correctly classified samples, and p CV-ANOVA = 0.041), **B** LGA vs AGA (R^2 = 0.996; Q^2 = 0.988, 100% correctly classified samples, and p CV-ANOVA = $2.57x10^{-15}$), **C** LGA vs SGA (R^2 = 0.990; Q^2 = 0.976, 100% correctly classified samples, and p CV-ANOVA = $9.20x10^{-13}$) represent the OPLS-DA models built for LC-MS ESI (+). **D** SGA vs AGA (R^2 = 0.998; Q^2 = 0.951, 100% correctly classified samples, and p CV-ANOVA = $2.75x10^{-7}$), **E** LGA vs AGA (R^2 = 0.997; Q^2 = 0.987, 100% correctly classified samples, and p CV-ANOVA = $6.72x10^{-16}$), **F** LGA vs SGA (R^2 = 0.997; Q^2 = 0.989, 100% correctly classified samples, and p CV-ANOVA = $1.82x10^{-18}$) represent the OPLS-DA models built for LC-MS ESI (−).

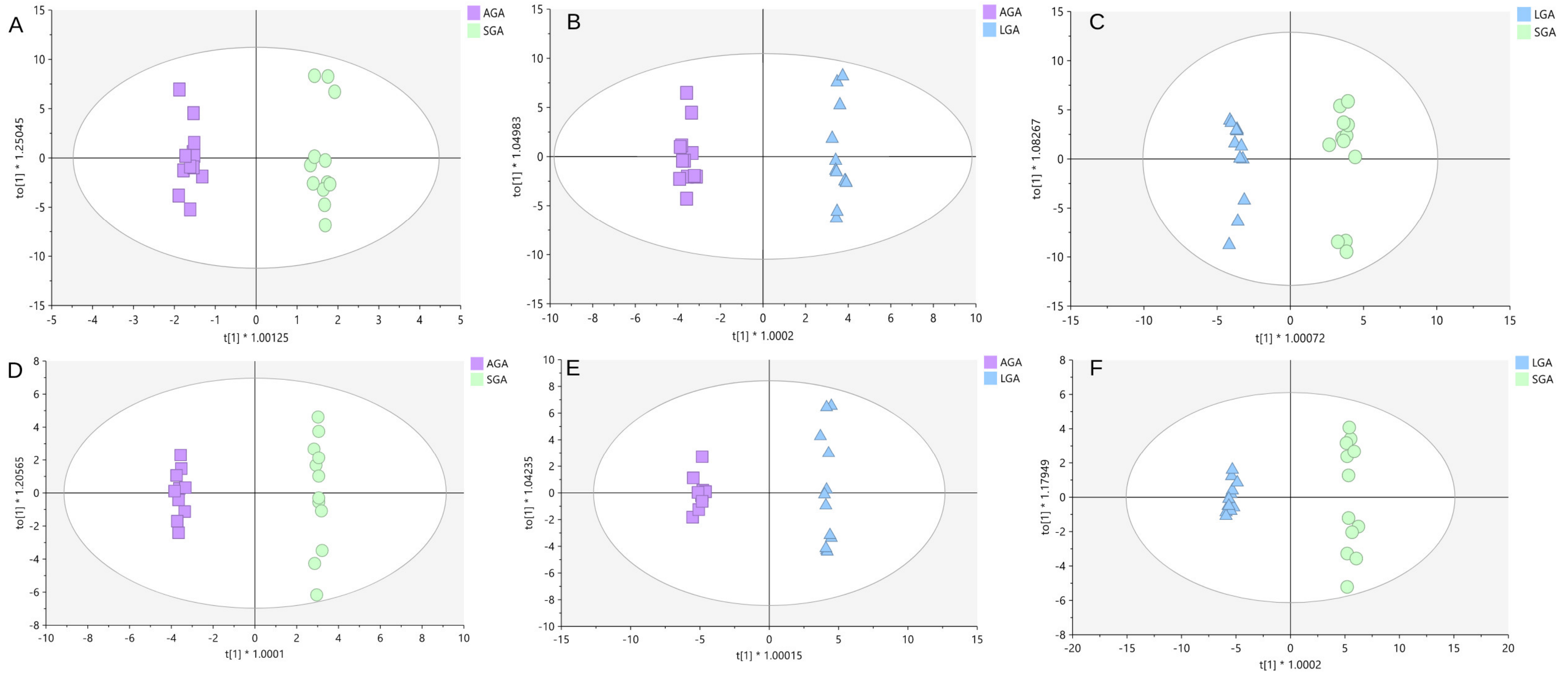

Supplement: Supplementary file 1 [file nutrients-14-03760-s001.zip › Supplementary Figure S2.pdf]
