# Supplementary material for: Birth Weight and Early Postnatal Outcomes: Association with the Cord Blood Lipidome
Source: Nutrients. 2022 Sep 12;14(18):3760. doi: 10.3390/nu14183760 (PMC9505183; doi:10.3390/nu14183760)
Supplement: Supplementary file 1 [file nutrients-14-03760-s001.zip › Supplementary File S1.pdf]

SUPPORTING INFORMATION

Weight at Birth & Early Postnatal Outcomes: Association with Cord Blood Lipidome

Carolina González[1*], Marcelo Santos[2*], Marta Díaz[2,3], Cristina García-Beltran[2,3], Carles Lerin[2], Coral Barbas[1], Lourdes Ibáñez[2,3#], David Sánchez-Infantes[4,5#]

[1] Centro de Metabolómica y Bioanálisis, Facultad de Farmacia, Universidad San Pablo-CEU, CEU Universities, Urbanización Montepríncipe, Boadilla del Monte, 28660 Madrid, Spain.

[2] Endocrinology Department, Institut de Recerca Sant Joan de Déu, Hospital Sant Joan de Déu, 08950 Barcelona (Spain)

[3] Biomedical Research Center (Red Fisiopatología de la Diabetes y enfermedades metabólicas) (CIBERDEM), ISCIII, Madrid, Spain.

[4] Centro de Investigación Biomédica en Red de Fisiopatología de la Obesidad y Nutrición (CIBERobn), Madrid, 28029, Spain

[5] Department of Health Sciences, Campus Alcorcón, University Rey Juan Carlos (URJC), E-28922 Madrid, Spain.

*Co-first

[#]Co-corresponding

Materials and methods

Reagents

Reverse-osmosed ultrapure water, used to prepare all the aqueous solutions, was obtained from a Milli-Qplus185 system (Millipore, Billerica, MA, USA). LC-MS grade methanol (MeOH), acetonitrile (ACN), and isopropanol (IPA) were obtained from Fisher Scientific (Pennsylvania, United States). methyl tert-butyl ether (MTBE) (HPLC grade, ≥99.8%), chloroform ($CHCl_3$) (HPLC grade, ≥99.9%) ammonium fluoride (NH_4F) (ACS reagent, ≥98%), and palmitic acid-d_{31} were purchased from Sigma-Aldrich (Steinheim, Germany). Analytical grade ammonia solution (28%, GPR RECTAPUR®), and acetic acid glacial (AnalaR® NORMAPUR®) were obtained from VWR Chemicals (Pennsylvania, United States). LightSPLASH® LIPIDOMIX® Quantitative Mass Spec Primary Standard, and sphinganine (d17:0) primary standard were obtained from Avanti Polar Lipids, Inc. (USA).

Liquid Chromatography Mass Spectrometry Analysis

The analysis of plasma extracts was performed as previously described[28]. For the analysis of the lipidomic profile, an Agilent 1290 Infinity II UHPLC system coupled to an Agilent 6546 quadrupole time-of-flight (QTOF) mass spectrometer was used. An Agilent InfinityLab Poroshell 120 EC-C18 (3.0 × 100 mm, 2.7 µm) (Agilent Technologies) column with a compatible guard column (Agilent InfinityLab Poroshell 120 EC-C18, 3.0 × 5 mm, 2.7 µm) thermostated at 50°C was used for the analysis. The autosampler injected a volume of 1 µL of each sample, which was maintained at 15°C to preserve compounds in a stable environment and avoid lipid precipitation.

The solvent system consisted of solvent (A) 10 mM ammonium acetate, 0.2 mM ammonium fluoride in 9:1 water/methanol and (B) 10 mM ammonium acetate, 0.2 mM ammonium fluoride in 2:3:5 acetonitrile/methanol/isopropanol. The chromatography gradient started at 70% of B at 0 – 1 min, 86% at 3.5 – 10 min, 100% B at 11-17 min. The starting conditions were recovered by minute 17, followed by a 2 min re-equilibration time; the total running time was 19 min. The flow rate was held constant, set at 0.6 mL/min. The multi-wash strategy consisted of a mixture of methanol:isopropanol (50:50, v/v) with the wash time set at 15 s, and aqueous phase: organic phase (30:70, v/v) mixture to assist in the starting conditions.

The Agilent 6546 QTOF mass spectrometer equipped with a dual AJS ESI ion source was set with the following parameters: 150 V fragmentor, 65 V skimmer, 3500 V capillary voltage, 750 V octopole radio frequency voltage, 10 L/min nebulizer gas flow, 200°C gas temperature, 50 psi nebulizer gas pressure, 12 L/min sheath gas flow, and 300°C sheath gas temperature. Data were collected in positive and negative ESI modes in separate runs, operated in full scan mode from 100 to 1700 *m/z* with a scan rate of 3 spectra/s. A solution consisting of two reference mass compounds were used throughout the whole analysis: purine ($C_5H_4N_4$) at *m/z* 121.0509 for the positive and *m/z* 119.0363 for the negative ionization modes; and HP-0921 ($C_{18}H_{18}O_6N_3P_3F_{24}$) at *m/z* 922.0098 for the positive and *m/z* 980.0163 (HP-0921 + acetate) for the negative ionization modes. These masses were continuously infused into the system to provide a constant mass correction.

Ten Iterative-MS/MS runs were performed using a QC sample for both ion modes at the end of the analytical run. They were operated with an MS and MS/MS scan rates of 3 spectra/s, 40-1700 *m/z* mass window, a narrow (~ 1.3 amu) MS/MS isolation width, 3 precursors per cycle, and 5000 counts and 0.001% of MS/MS threshold. Five iterative-MS/MS runs were set with a collision energy of 20 eV, and the subsequent five runs were performed at 40 eV. References masses and contaminants detected in blank samples were excluded from the analysis to avoid inclusion in the iterative-MS/MS[28].

Lipid Annotation

Correctly annotating the lipid species and additional compounds detected by LC-MS is crucial to provide a proper biological explanation for the results obtained. The annotation workflow consisted of three steps: initial tentative identification of lipid features, based on the MS1 data, using our online tool CEU Mass Mediator (CMM) (http://ceumass.eps.uspceu.es/mediator/) [30,31]. This tool for mass-based compound annotation comprises the information available in different databases (KEGG, HMDB, LIPID MAPS, METLIN, MINE, and an in-house library). This stage started with the tentative assignment based on (i) accurate mass (maximum mass error tolerance 10 ppm); (ii) retention time (RT); (iii) isotopic pattern distribution; (iv) possibility of cation and anion formation; and (v) adduct formation pattern.

Secondly, the raw LC-MS/MS data obtained was imported into the Lipid Annotator software (Agilent Technologies Inc., Santa Clara, CA, USA) to increase the level of confidence annotation. The software builds a fragmentation-based (MS/MS) library comprising the m/z of all the precursors identified as lipids by the software, together with their corresponding RT. The Lipid Annotator method [56] was set as follows: ion species $[M+H]^+$, $[M+Na]^+$, and $[M+NH_4]^+$ for positive; and $[M-H]^-$, and $[M+CH_3COOH-H]^-$ for negative ionization mode. Then, for both ion modes, the Q-Score was set at ≥ 50; all the lipid classes were selected, mass deviation was established as ≤ 20 ppm, fragment score threshold was fixed as ≥ 30, and total score was set at ≥ 60.

Finally, a manual MS/MS spectral interpretation was carried out using the software Agilent MassHunter Qualitative (version 10.0), comparing the retention time and MS/MS fragmentation to the available spectral data included in METLIN [32], MS-DIAL[33], and LIPID MAPS [34]. The software ChemSketch MS Fragmenter (ACD/Laboratories, v.2015.2.5) was also employed for product ion and fragment structure prediction. The MS/MS analysis of both IS and the LightSPLASH® Lipidomix® standard mixture also provided higher confidence level annotations for the lipid classes included in the mixture.

Quantification and Semi-quantification

One of the most significant challenges when performing a non-targeted lipidomics-based approach is the quantification of the analytes of interest. The addition of the LightSPLASH® Lipidomix® standard mixture, which contains non-endogenous class-specific lipid standards, together with the sphinganine (d17:0), and palmitic acid-d_{31} allowed the

quantification of 15 lipid classes by assuming that each lipid standard has the same response factor (RF) as the lipid of interest belonging to that class [57].

The remaining lipids and metabolites detected as significantly affected were semi-quantified using the standard presenting the minimum difference in RT and in mass versus the biological compound [57].

References

28. Gonzalez-Riano, C.; Gradillas, A.; Barbas, C. Exploiting the Formation of Adducts in Mobile Phases with Ammonium Fluoride for the Enhancement of Annotation in Liquid Chromatography-High Resolution Mass Spectrometry Based Lipidomics. *Journal of Chromatography Open* **2021**, *1*, 100018, doi:10.1016/J.JCOA.2021.100018.

30. Gil-de-la-Fuente, A.; Godzien, J.; Saugar, S.; Garcia-Carmona, R.; Badran, H.; Wishart, D.S.; Barbas, C.; Otero, A. CEU Mass Mediator 3.0: A Metabolite Annotation Tool. *J Proteome Res* **2019**, *18*, 797–802, doi:10.1021/acs.jproteome.8b00720.

31. Gil de la Fuente, A.; Godzien, J.; Fernández López, M.; Rupérez, F.J.; Barbas, C.; Otero, A. Knowledge-Based Metabolite Annotation Tool: CEU Mass Mediator. *J Pharm Biomed Anal* **2018**, *154*, 138–149, doi:10.1016/j.jpba.2018.02.046.

32. Smith, C.A.; O'Maille, G.; Want, E.J.; Qin, C.; Trauger, S.A.; Brandon, T.R.; Custodio, D.E.; Abagyan, R.; Siuzdak, G. METLIN: A Metabolite Mass Spectral Database. *Ther Drug Monit* **2005**, *27*, 747–751, doi:10.1097/01.ftd.0000179845.53213.39.

33. Tsugawa, H.; Ikeda, K.; Takahashi, M.; Satoh, A.; Mori, Y.; Uchino, H.; Okahashi, N.; Yamada, Y.; Tada, I.; Bonini, P.; et al. MS-DIAL 4: Accelerating Lipidomics Using an MS/MS, CCS, and Retention Time Atlas. *BioRxiv* **2020**, 1159–1163.

34. Sud, M.; Fahy, E.; Cotter, D.; Brown, A.; Dennis, E.A.; Glass, C.K.; Merrill, A.H.; Murphy, R.C.; Raetz, C.R.H.; Russell, D.W.; et al. LMSD: LIPID MAPS Structure Database. *Nucleic Acids Res* **2007**, *35*, D527-32, doi:10.1093/nar/gkl838.

56. Koelmel, J.P.; Li, X.; Stow, S.M.; Sartain, M.J.; Murali, A.; Kemperman, R.; Tsugawa, H.; Takahashi, M.; Vasiliou, V.; Bowden, J.A.; et al. Lipid Annotator: Towards Accurate Annotation in Non-Targeted Liquid Chromatography High-Resolution Tandem Mass Spectrometry (LC-HRMS/MS) Lipidomics Using A Rapid and User-Friendly Software. *Metabolites* **2020**, *10*, doi:10.3390/metabo10030101.

57. Pieke, E.N.; Granby, K.; Trier, X.; Smedsgaard, J. A Framework to Estimate Concentrations of Potentially Unknown Substances by Semi-Quantification in Liquid Chromatography Electrospray Ionization Mass Spectrometry. *Analytica Chimica Acta* **2017**, *975*, 30–41, doi:10.1016/J.ACA.2017.03.054.
