# Supplementary material for: Birth Weight and Early Postnatal Outcomes: Association with the Cord Blood Lipidome
Source: Nutrients. 2022 Sep 12;14(18):3760. doi: 10.3390/nu14183760 (PMC9505183; doi:10.3390/nu14183760)
Supplement: Supplementary file 1 [file nutrients-14-03760-s001.zip › Supplementary Table S1.pdf]

Table S1. Internal standards (IS) of the LightSPLASH® Lipidomix® Mass Spec Standard (in MeOH), and the additional IS (in MeOH) standards added to each sample.

Mixture Component	Monoisotopic mass	RT (min)	Adduct ESI(+) / ESI (-)	Final concentration in sample (µg/mL)	CV (%) ESI(+) / ESI(-)
PC(15:0/18:1)	745.5621	7.91	$[M+H]^+$ / $[M+CH_3COOH-H]^-$	0.125	2.7 / 5.1
LPC(18:1)	521.3481	2.65	$[M+H]^+$ / $[M+CH_3COOH-H]^-$	0.125	2.3 / 2.7
PE(15:0/18:1)	703.5152	8.30	$[M+H]^+$ / $[M-H]^-$	0.125	2.8 / 3.7
LPE(18:1)	479.3011	2.76	$[M+H]^+$ / $[M-H]^-$	0.125	3.2 / 7.4
PG(15:0/18:1)	734.5097	6.41	$[M+NH_4]^+$ / $[M-H]^-$	0.125	4.0 / 3.4
PI(15:0/18:1)	822.5258	6.18	$[M+NH_4]^+$ / $[M-H]^-$	0.125	6.1 / 3.2
PS(15:0/18:1)	747.5050	6.34	$[M+H]^+$ / $[M-H]^-$	0.125	5.3 / 5.3
TG(15:0/18:1/15:0)	804.7206	13.90	$[M+NH_4]^+$ / N.D.	0.125	6.7 / ND
DG(15:0/18:1)	580.5066	11.61	$[M+NH_4]^+$ / $[M+CH_3COOH-H]^-$	0.125	10.2 / 3.7
MG(18:1)	356.2926	3.60	$[M+Na]^+$ / N.D.	0.125	3.1 / ND
CE(18:1)	650.6002	15.31	$[M+NH_4]^+$ / N.D.	0.125	6.2 / ND
SM(d18:1/18:1)	728.5832	7.16	$[M+H]^+$ / $[M+CH_3COOH-H]^-$	0.125	5.8 / 6.4
Cer(d18:1/15:0)	523.4964	7.70	$[M+H]^+$ / $[M+CH_3COOH-H]^-$	0.125	4.9 / 2.2
Sphinganine (d17:0)	287.2824	1.79	$[M+H]^+$ / $[M+CH_3COOH-H]^-$	1.000	5.9 / 6.0
Palmitic acid-d_{31}	287.4348	3.12	N.D. / $[M-H]^-$	3.750	- / 1.7

RT, retention time; ESI, electrospray ionization; CV, coefficient of variation.
