# Supplementary material for: Birth Weight and Early Postnatal Outcomes: Association with the Cord Blood Lipidome
Source: Nutrients. 2022 Sep 12;14(18):3760. doi: 10.3390/nu14183760 (PMC9505183; doi:10.3390/nu14183760)
Supplement: Supplementary file 1 [file nutrients-14-03760-s001.zip › Supplementary Table S2.pdf]

Supplementary Table S2. Data from infants born small- (SGA), appropriate (AGA) or large-for-gestational-age (LGA) and from their mothers

	SGA (n=12) 50% girls	AGA (n=12) 58% girls	LGA (n=12) 50% girls	*P value*
**Maternal data**				
Age at conception (yr)	32.2 ± 1.4	30.4 ± 1.3	33.6 ± 1.1	0.2405
Pre-gestational BMI (kg/m^2)	23.3 ± 0.7	22.8 ± 0.8	30.5 ± 1.8	<0.0001
BMI at the end of pregnancy	28.3 ± 0.7	27.8 ± 1.0	35.8 ± 1.4	<0.0001
Gestational age (wk)	38.6 ± 0.4	39.6 ± 0.4	40.2 ± 0.3	0.0053
**Infants' data**				
**At birth**				
Anthropometric & Endocrine-metabolic variables				
Weight (kg)	2.3 ± 0.1	3.3 ± 0.1	4.4 ± 0.1	<0.0001
Length (cm)	45.7 ± 0.5	49.9 ± 0.3	53.0 ± 0.4	<0.0001
BMI Z-score	-2.2 ± 0.2	0.0 ± 0.2	1.6 ± 0.2	<0.0001
HOMA-IR	0.9 ± 0.3	1.8 ± 0.6	0.9 ± 0.4	0.3074
IGF-I (ng/ml)	51 ± 6	78 ± 8	48 ± 8	0.0110
HMW-adip (mg/ml)	31.2 ± 6.1	31.9 ± 3.2	31.1 ± 3.8	0.9881
*Body composition variables**				
Fat mass (gr)	417 ± 56	627 ± 92	1267 ± 111	<0.0001
Abdominal fat (gr)	22 ± 4	35 ± 7	73 ± 8	<0.0001
Lean mass (gr)	2485 ± 122	3019 ± 80	3542 ± 86	<0.0001
**At 4 months**				
Anthropometric & Endocrine-metabolic variables				
Weight (kg)	5.8 ± 0.3	6.9 ± 0.2	7.9 ± 0.2	<0.0001
Length (cm)	60.2 ± 0.9	62.2 ± 0.8	65.9 ± 0.5	0.0001
BMI Z-score	-0.7 ± 0.3	0.6 ± 0.3	0.8 ± 0.2	0.0006
HOMA-IR	2.6 ± 0.7	1.4 ± 0.2	0.4 ± 0.1	0.0040
IGF-I (ng/ml)	78 ± 8	57 ± 8	30 ± 3	0.0002
HMW-adip (mg/ml)	32.7 ± 5.7	23.8 ± 2.1	37.7 ± 5.2	0.0866
Body composition variables				
Fat mass (gr)	2054 ± 147	2767 ± 201	3085 ± 210	0.0028
Abdominal fat (gr)	129 ± 11	169 ± 16	195 ± 19	0.0252
Lean mass (gr)	4125 ± 219	4407 ± 190	5068 ± 194	0.0095

**At 12 months**				
Anthropometric & Endocrine-metabolic variables				
Weight, kg	9.0 ± 0.3	10.6 ±0.4	11.2 ± 0.3	0.0004
Length, cm	73.3 ± 0.8	76.8 ± 1.1	77.8 ± 0.9	0.0047
BMI Z-score	0.8 ± 0.4	1.0 ± 0.3	1.2 ± 0.2	0.0429
HOMA-IR	0.8 ± 0.2	1.5 ± 0.4	0.3 ± 0.2	0.0512
IGF-I (ng/ml)	94 ± 16	89 ± 12	37 ± 5	0.0055
HMW-adip (mg/ml)	22.6 ± 2.0	17.3 ± 2.0	15.6 ± 8.6	0.1722
Body composition variables				
Fat mass (gr)	3393 ± 158	4200 ± 271	3791 ± 271	0.0649
Abdominal fat (gr)	165 ± 12	250 ± 29	229 ± 21	0.0242
Lean mass (gr)	5744 ± 219	6810 ± 270	7716 ± 226	<0.0001

BMI, body mass index; HOMA-IR, homeostasis model assessment insulin resistance; IGF-I, insulin-like growth factor I; HMW-adip, high-molecular-weight adiponectin.

Values are mean ± standard error. P-values by linear-trend ANOVA.

*At age 15 days
