# Supplementary material for: Birth Weight and Early Postnatal Outcomes: Association with the Cord Blood Lipidome
Source: Nutrients. 2022 Sep 12;14(18):3760. doi: 10.3390/nu14183760 (PMC9505183; doi:10.3390/nu14183760)
Supplement: Supplementary file 1 [file nutrients-14-03760-s001.zip › Supplementary Table S3.pdf]

Table S3. Cord plasma lipids in newborns born small- (SGA, n=12), appropriate (AGA, n=12) or large-for-gestational-age (LGA, n=12). Those lipids showing statistically significant differences for any of the tested comparisons among study subgroups are shown.

							LGA vs AGA				SGA vs AGA				LGA vs SGA				*Semi-quantification in ppm*		
Name	*m/z*	RT (min)	Formula	CV QC	p kruskal	p kruskal BH	FC	*p* value	*p*BH	VIP	FC	*p* value	*p*BH	VIP	FC	*p* value	*p*BH	VIP	ppm SGA (0.12ppm)	ppm LGA (0.12ppm)	ppm AGA (0.12ppm)
**Fatty Acyls**																					
Eicosanoids																					
FA 20:4;O3 (Lipoxin B4)	351.218	0.95	C20H32O5	4.0	0.075	0.26	3.11	0.021	0.083	1.33	1.70	0.096	0.19	1.14	1.83	0.28	0.32	0.58	0.13	0.24	0.076
1(3)-glyceryl-6-keto-PGF1alpha / 2-glyceryl-6-keto-PGF1α	443.265	0.84	C23H40O8	2.8	4.9E-06	1.4E-04	-LGA	-	-	4.40	5.41	0.20	0.26	1.73	-LGA	-	-	4.07	0.26	0	0.049
11-HEDE	301.199	0.89	C20H36O3	8.6	0.0051	0.051	0.35	0.77	0.95	0.61	2.99	0.0047	0.045	2.04	0.12	3.2E-04	0.0029	1.75	0.076	0.0089	0.025
12,20-DiHETE	355.228	2.52	C20H32O4	3.9	6.9E-06	1.4E-04	-LGA	-	-	4.61	1.59	0.83	0.89	1.25	-LGA	-	-	3.97	0.17	0	0.11
12-HETE	339.233	3.21	C20H32O3	1.1	0.0017	0.018	0.01	0.011	0.063	2.47	0.87	0.97	0.99	1.36	0.01	5.9E-04	0.0043	2.50	4.6	0.055	5.3
15-HpETE	335.223	0.96	C20H32O4	3.7	0.15	0.37	4.70	0.040	0.12	1.66	3.04	0.16	0.22	1.64	1.55	0.51	0.52	0.67	0.16	0.24	0.05
19-hydroxy-PGE2	429.25	0.82	C20H34O6	5.4	2.2E-07	1.4E-05	-LGA	-	-	3.41	22.20	8.7E-05	0.0025	3.45	-LGA	-	-	3.84	0.061	0	0.0027
5S-HpEPE	353.212	1.71	C20H30O4	2.8	6.9E-06	1.4E-04	-LGA	-	-	4.76	0.99	0.67	0.75	0.22	-LGA	-	-	4.15	0.18	0	0.18
Fatty Acids and Conjugates																					
α-Linolenic acid	277.217	2.55	C18H30O2	2.4	0.080	0.26	0.86	0.34	0.58	0.28	1.25	0.16	0.22	0.67	0.68	0.045	0.072	0.64	0.5	0.4	0.4
Docosatetraenoic acid (Adrenic Acid)	331.264	3.65	C22H36O2	1.4	0.013	0.094	0.91	0.18	0.37	0.30	1.18	0.093	0.19	0.65	0.76	0.0194*	0.019	0.56	0.4	0.3	0.3
Docosahexaenoic acid (DHA)	327.233	2.77	C22H32O2	2.1	0.0057	0.054	0.97	0.83	0.97	0.14	1.55	0.0058	0.045	1.07	0.63	0.020*	0.020	0.75	2.2	1.4	1.4
Docosapentaenoic acid (DPA)	329.249	3.16	C22H34O2	5.8	0.030	0.16	1.03	0.76	0.95	0.13	1.56	0.019	0.077	0.99	0.66	0.026*	0.026	0.61	0.1	0.1	0.1
Eicosadienoic acid	307.264	3.84	C20H36O2	2.4	0.053	0.23	0.91	0.37	0.61	0.16	1.18	0.14	0.23	0.67	0.77	0.022*	0.022	0.56	0.3	0.3	0.3
Hexacosanedioic acid	425.364	3.52	C26H50O4	2.4	9.4E-05	0.0016	0.67	9.0E-04	0.037	0.96	1.36	0.021	0.10	0.91	0.49	0.0027*	0.0027	1.07	0.3	0.1	0.2
Linoleic acid	279.233	3.05	C18H32O2	1.5	0.037	0.19	0.80	0.37	0.61	0.32	1.40	0.081	0.18	0.87	0.57	0.022*	0.022	0.81	10.0	5.7	7.1
Myristic acid	227.202	2.56	C14H28O2	3.2	0.13	0.36	0.91	0.45	0.67	0.16	1.22	0.21	0.28	0.68	0.75	0.054*	0.054	0.57	1.3	1.0	1.1
Oleic acid	281.249	3.63	C18H34O2	1.1	0.058	0.23	0.95	1.00	1.00	0.05	1.41	0.070	0.17	0.94	0.68	0.016	0.032	0.59	0.3	0.2	0.2
Palmitic acid	255.233	3.46	C16H32O2	2.4	0.11	0.30	0.98	0.85	0.94	0.10	1.26	0.074	0.16	0.74	0.78	0.045	0.072	0.48	22.96	17.80	18.21
Fatty acyl carnitines																					
L-carnitine	162.112	0.74	C7H15NO3	5.3	0.060	0.075	1.29	0.015	0.14	0.39	1.07	0.50	0.53	0.30	1.20	0.090	0.17	0.30	0.026	0.031	0.024
Decanoylcarnitine	316.248	0.92	C17H33NO4	7.9	0.045	0.067	1.30	0.027	0.17	0.38	1.50	0.033	0.052	1.12	0.87	0.36	0.49	0.15	0.00051	0.00044	0.00034
Decenoylcarnitine	314.233	0.86	C17H31NO4	8.6	0.034	0.061	1.31	0.039	0.18	0.38	1.49	0.025	0.045	1.12	0.88	0.58	0.72	0.09	0.00047	0.00041	0.00031
Octenoylcarnitine	286.201	0.79	C15H27NO4	9.8	0.35	0.35	1.25	0.14	0.27	0.30	1.26	0.28	0.32	0.64	0.99	0.85	0.92	0.20	0.00032	0.00032	0.00026
Oleoylcarnitine	426.358	2.43	C25H47NO4	5.5	0.0091	0.043	1.46	0.0035	0.092	0.65	1.15	0.22	0.26	0.49	1.27	0.038	0.10	0.40	0.00085	0.0011	0.00074
Palmitoylcarnitine	400.342	2.25	C23H45NO4	5.4	0.0017	0.018	1.46	2.8E-04	0.029	0.64	1.18	0.14	0.16	0.48	1.24	0.029	0.083	0.38	0.00083	0.0010	0.00070
Fatty esters																					
FAHFA(18:1-(10-O-16:0)	595.494	3.8	C34H64O4	1.4	0.0014	0.016	0.71	0.0076	0.048	0.75	1.33	0.082	0.16	0.83	0.54	0.0014	0.0077	0.94	0.024	0.013	0.018
FAHFA(30:1)	539.431	2.95	C30H56O4	2.6	1.1E-04	0.0017	0.65	0.0022	0.037	0.92	1.36	0.041	0.11	0.92	0.47	1.2E-05	4.E-04	1.10	0.049	0.023	0.036
Ascorbyl palmitate	473.275	0.85	C22H38O7	4.1	1.8E-07	1.4E-05	-	-	-	-	+SGA	-	-	5.49	-LGA	-	-	3.80	0.051	0	0
3-Hexenyl 3-hydroxybutanoate; WE 10:1;O	245.14	0.78	C10H18O3	8.1	1.8E-07	1.4E-05	-	-	-	-	+SGA	-	-	4.80	-LGA	-	-	3.39	0.11	0	0
Octadecanoids																					
9-HODE	297.242	12.88	C18H32O3	7.1	0.0033	0.021	3.55	0.045	0.15	1.56	1.98	0.0032	0.013	0.96	1.79	0.62	0.74	1.11	0.0091	0.016	0.0046
9-OxoODE	295.227	12.71	C18H30O3	3.1	0.079	0.094	3.17	0.044	0.15	1.88	2.49	0.028	0.11	2.18	1.27	0.98	0.99	1.58	0.032	0.041	0.013
Oxo fatty acids																					
FA 12:1;O	273.17	0.76	C12H22O3	4.0	0.27	0.53	0.90	0.50	0.69	0.30	8.21	0.31	0.36	1.80	0.11	0.060	0.084	1.34	0.019	0.0021	0.0023
FA 26:2;O	467.374	2.72	C26H48O3	2.3	5.2E-04	0.0069	0.69	0.0033	0.048	0.90	1.17	0.13	0.20	0.55	0.58	2.4E-04	0.0030	0.90	1.4	0.82	1.2
FA 28:2;O	495.405	3.78	C28H52O3	4.2	0.0012	0.014	0.70	0.0070	0.048	0.82	1.28	0.093	0.19	0.80	0.55	0.0011	0.0071	0.97	0.31	0.17	0.24
methyl-FA 18:3;2OOH	415.236	0.81	C19H32O6	5.1	1.3E-06	6.7E-05	-LGA	-	-	3.74	8.42	0.0062	0.045	2.52	-LGA	-	-	3.82	0.096	0	0.011
6-Hydroxypentadecanedioic acid	287.187	0.84	C15H28O5	6.1	5.5E-06	1.4E-04	-LGA	-	-	3.93	4.83	0.28	0.33	1.73	-LGA	-	-	3.66	0.061	0	0.013
**Glycerolipids**																					
Diacylglycerols																					
DG 16:0/18:1/0:0	612.556	11.83	C37H70O5	9.0	0.095	0.11	1.50	0.048	0.18	0.75	1.32	0.11	0.14	0.80	1.14	0.49	0.59	0.51	0.033	0.037	0.025
DG 16:0/18:2/0:0	610.539	11.34	C37H68O5	6.0	1.5E-05	0.0015	1.45	0.053	0.20	0.70	3.55	1.8E-04	0.011	3.46	0.41	4.8E-04	0.0083	1.33	0.041	0.017	0.012
DG 18:1/18:1/0:0	638.572	11.87	C39H72O5	5.9	0.064	0.078	1.27	0.027	0.17	0.39	1.15	0.033	0.052	0.41	1.11	0.34	0.47	0.28	0.079	0.087	0.068
DG 18:1/18:2/0:0	636.556	11.54	C39H70O5	5.8	0.046	0.067	1.22	0.078	0.20	0.38	1.32	0.012	0.026	0.78	0.93	0.47	0.61	0.34	0.075	0.070	0.057
DG 18:2/16:0/0:0	651.52	11.58	C37H68O5	2.6	0.30	0.57	1.31	0.27	0.49	0.75	1.56	0.047	0.12	1.06	0.84	0.46	0.48	0.57	0.0038	0.0059	0.0050
DG 18:0/18:2/0:0	655.507	11.94	C39H72O5	7.9	0.076	0.26	1.52	0.026	0.10	0.91	1.28	0.077	0.16	0.82	1.19	0.31	0.35	0.52	0.0015	0.0020	0.0023
DG 18:3/19:0/0:0	677.537	11.66	C39H70O5	1.6	0.050	0.23	1.26	0.22	0.44	0.65	1.48	0.0095	0.060	1.05	0.85	0.24	0.28	0.52	0.0065	0.0055	0.0044
Triacylglycerols																					
TG 14:0/18:1/22:6	915.683	13.2	C57H96O6	4.9	0.034	0.061	0.98	0.88	0.93	0.25	1.63	0.013	0.027	1.30	0.60	0.013	0.055	0.79	0.010	0.006	0.006
TG 14:0/22:2/18:2	921.731	13.94	C57H102O6	4.8	0.024	0.057	1.20	0.078	0.20	0.31	1.29	0.0061	0.021	0.71	0.93	0.62	0.74	0.29	0.028	0.026	0.022
TG 14:0/18:3/22:3	917.7	13.57	C57H98O6	6.7	0.023	0.057	0.86	0.18	0.32	0.27	1.80	0.061	0.087	1.46	0.48	0.016	0.068	1.00	0.0041	0.0020	0.0023
TG 14:0/22:4/18:2	925.725	13.23	C59H98O6	5.5	0.056	0.073	0.91	0.36	0.49	0.34	1.51	0.042	0.063	1.09	0.60	0.022	0.070	0.80	0.0023	0.0014	0.0015
TG 15:0/18:3/19:0	895.715	13.94	C55H100O6	6.0	0.016	0.049	1.32	0.056	0.19	0.48	1.41	0.0071	0.020	0.93	0.94	0.65	0.75	0.40	0.061	0.057	0.043
TG 15:0/20:0/O-18:0	901.802	13.55	C57H98O6	14.7	0.017	0.049	1.15	0.21	0.34	0.27	1.48	0.0050	0.019	1.05	0.78	0.068	0.15	0.42	0.0073	0.0057	0.0050
TG 16:0/16:0/20:4	872.77	13.55	C55H98O6	4.3	0.017	0.049	1.27	0.21	0.33	0.42	1.80	0.0049	0.019	1.54	0.70	0.095	0.19	0.66	0.080	0.056	0.045
TG 16:0/16:1/20:4	870.766	13.94	C55H96O6	5.0	0.035	0.061	1.54	0.061	0.20	0.64	1.69	0.0076	0.020	1.36	0.91	0.51	0.65	0.57	0.0035	0.0032	0.0021
TG 16:0/16:1/22:6	894.754	13.19	C57H96O6	6.2	0.039	0.061	1.00	0.76	0.81	0.29	1.77	0.018	0.034	1.45	0.56	0.030	0.086	0.91	0.014	0.008	0.008
TG 16:0/17:2/22:2	914.816	13.94	C58H104O6	24.3	0.048	0.067	1.41	0.052	0.18	0.53	1.46	0.019	0.035	0.98	0.97	0.84	0.89	0.47	0.041	0.040	0.028
TG 16:0/18:0/18:3	879.741	13.94	C55H100O6	3.8	0.014	0.047	1.47	0.054	0.20	0.59	1.56	0.0080	0.020	1.18	0.94	0.59	0.73	0.50	0.010	0.009	0.006

Name	m/z	RT (min)	Formula	CV QC	p kruskal	p kruskal BH	LGA vs AGA				SGA vs AGA				LGA vs SGA				Semi-quantification in ppm		
							FC	*p* value	*p*BH	VIP	FC	*p* value	*p*BH	VIP	FC	*p* value	*p*BH	VIP	ppm SGA (0.12ppm)	ppm LGA (0.12ppm)	ppm AGA (0.12ppm)
TG 16:0/18:1/18:1	876.8	13.56	C55H102O6	8.8	0.030	0.059	1.08	0.48	0.56	0.19	1.40	0.012	0.026	0.95	0.77	0.052	0.12	0.45	0.028	0.021	0.020
TG 16:0/18:1/18:3	877.725	13.55	C55H98O6	5.1	0.014	0.047	1.23	0.14	0.27	0.36	1.58	0.0056	0.019	1.22	0.78	0.15	0.26	0.52	0.0054	0.0042	0.0034
TG 16:0/18:1/20:4	898.785	13.56	C57H100O6	16.5	0.055	0.073	1.10	0.47	0.50	0.25	1.53	0.018	0.035	1.21	0.72	0.052	0.12	0.56	0.035	0.025	0.023
TG 16:0/18:1/22:6	922.788	13.56	C59H100O6	7.3	0.021	0.055	1.02	0.87	0.93	0.19	1.69	0.0071	0.020	1.38	0.60	0.0091	0.055	0.78	0.044	0.026	0.026
TG 16:0/18:2/20:4	896.77	13.24	C57H98O6	7.5	0.027	0.058	1.01	0.93	0.95	0.08	1.42	0.026	0.045	0.99	0.71	0.0096	0.055	0.51	0.019	0.014	0.013
TG 16:0/18:2/22:6	920.769	13.23	C59H98O6	2.6	0.025	0.057	0.91	0.43	0.53	0.40	1.88	0.020	0.037	1.59	0.48	0.013	0.055	1.08	0.030	0.014	0.016
TG 16:0/18:3/22:5	941.7	13.23	C59H98O6	6.7	0.037	0.061	0.84	0.34	0.47	0.69	2.15	0.033	0.052	1.83	0.39	0.019	0.067	1.42	0.011	0.004	0.005
TG 16:0/20:1/O-18:0	913.796	13.55	C57H110O5	22.9	0.037	0.061	1.27	0.14	0.27	0.39	1.64	0.012	0.026	1.30	0.77	0.18	0.29	0.60	0.036	0.028	0.022
TG 16:0/20:4/22:6	944.771	13.23	C61H98O6	21.1	0.056	0.073	0.90	0.57	0.49	0.58	2.25	0.049	0.074	1.97	0.40	0.022	0.070	1.34	0.0088	0.0035	0.0039
TG 16:1/18:1/18:1	874.786	13.94	C55H100O6	2.8	0.024	0.057	1.41	0.081	0.20	0.57	1.52	0.011	0.024	1.12	0.93	0.57	0.72	0.49	0.18	0.17	0.12
TG 16:1/18:1/18:2	872.764	13.54	C55H98O6	6.9	0.0098	0.044	1.33	0.035	0.20	0.38	1.53	0.0045	0.020	1.03	0.87	0.16	0.26	0.46	0.019	0.016	0.012
TG 16:1/18:1/20:2	905.757	13.94	C57H102O6	7.0	0.014	0.047	1.35	0.030	0.15	0.46	1.42	0.0061	0.021	1.02	0.95	0.71	0.79	0.36	0.0041	0.0039	0.0029
TG 16:1/18:2/20:3	896.77	13.56	C57H98O6	15.7	0.014	0.047	1.16	0.22	0.36	0.28	1.60	0.0058	0.019	1.24	0.73	0.051	0.12	0.59	0.051	0.037	0.032
TG 16:0/20:5/22:6	942.753	12.81	C61H96O6	11.2	0.21	0.22	1.21	0.44	0.53	0.63	2.58	0.040	0.062	2.51	0.47	0.13	0.24	1.06	0.0014	0.0006	0.0005
TG 18:1/18:1/18:4	917.699	13.4	C57H98O6	7.4	0.042	0.063	0.85	0.10	0.24	0.33	1.26	0.12	0.14	0.70	0.67	0.021	0.069	0.64	0.017	0.012	0.014
TG 18:0/18:3/18:4	915.683	13.19	C57H96O6	5.1	0.051	0.069	0.95	0.62	0.70	0.31	1.53	0.037	0.056	1.12	0.62	0.027	0.081	0.79	0.010	0.006	0.006
TG 18:0/18:3/20:4	922.777	13.35	C59H100O6	30.2	0.091	0.10	1.46	0.16	0.29	0.69	2.03	0.030	0.050	2.07	0.72	0.26	0.38	0.64	0.0049	0.0035	0.0024
TG 18:1/18:1/18:2	900.797	13.56	C57H102O6	13.8	0.011	0.044	1.09	0.41	0.53	0.19	1.61	0.0013	0.013	1.30	0.68	0.0082	0.055	0.60	0.022	0.015	0.014
TG 18:1/18:2/18:1	900.801	13.94	C57H102O6	7.9	0.040	0.063	1.20	0.12	0.25	0.32	1.37	0.010	0.024	0.88	0.88	0.29	0.43	0.37	0.075	0.066	0.055
TG 18:1/18:2/18:4	894.754	13.24	C57H96O6	5.5	0.035	0.061	1.04	0.62	0.70	0.12	1.45	0.0057	0.019	1.03	0.72	0.013	0.055	0.52	0.021	0.015	0.014
TG 18:1/18:3/18:3	894.752	13.37	C57H96O6	4.2	0.046	0.067	0.94	0.42	0.53	0.17	1.28	0.036	0.055	0.71	0.73	0.013	0.055	0.52	0.036	0.027	0.028
TG 18:1/18:2/22:0	958.878	13.94	C61H112O6	12.7	0.0064	0.035	1.32	0.013	0.14	0.44	1.38	0.0038	0.019	0.87	0.95	0.67	0.76	0.31	0.0017	0.0016	0.0012
TG 20:4/16:0/O-18:0	907.752	12.44	C57H104O5	4.6	0.0011	0.014	2.88	0.0017	0.057	1.45	2.50	0.0017	0.014	2.47	1.15	0.89	0.94	0.90	0.0020	0.0022	0.0008
TG 80:5	1311.17	14.11	C83H156NO9	10.8	0.031	0.060	0.86	0.16	0.29	0.96	0.49	0.0014	0.014	2.27	1.76	0.41	0.55	0.63	0.00024	0.00042	0.00049
TG 84:5	1367.23	14.69	C87H164NO9	7.7	0.022	0.056	0.73	0.085	0.22	0.80	0.63	0.0031	0.018	1.23	1.15	0.46	0.57	0.31	0.00040	0.00047	0.00064
**Glycerophospholipids**																					
Glycerophosphocholines																					
PC O-16:1/22:6	790.575	7.77	C46H80NO7P	6.7	0.036	0.061	0.64	0.0069	0.14	0.88	0.95	0.46	0.50	0.41	0.67	0.034	0.096	0.74	0.0070	0.0047	0.0073
PC O-18:1/18:2	770.605	9.31	C44H84NO7P	16.1	0.027	0.058	0.82	0.089	0.21	0.43	0.70	0.0055	0.019	0.96	1.18	0.71	0.80	0.19	0.0055	0.0065	0.0079
PC O-22:1/22:3	880.715	12.13	C52H98NO7P	28.1	0.031	0.060	1.36	0.14	0.27	0.57	1.68	0.012	0.026	1.79	0.81	0.23	0.35	0.42	0.028	0.023	0.017
PC O-18:1/0:0	508.376	3.05	C26H54NO6P	5.0	0.030	0.059	1.45	0.030	0.15	0.60	0.99	0.61	0.63	0.43	1.47	0.026	0.083	0.63	0.0027	0.0040	0.0027
LPC 0:0/16:0	496.34	2.28	C24H50NO7P	4.9	0.041	0.063	1.23	0.13	0.26	0.31	0.91	0.23	0.26	0.42	1.35	0.019	0.070	0.46	0.023	0.031	0.026
LPC 0:0/18:0	524.371	3.13	C26H54NO7P	9.6	0.058	0.073	1.36	0.019	0.15	0.45	1.17	0.34	0.37	0.43	1.16	0.19	0.32	0.37	0.0094	0.0109	0.0080
LPC 14:0/0:0	468.308	1.68	C22H46NO7P	5.6	0.0046	0.028	1.07	0.43	0.53	0.13	0.76	0.0019	0.014	0.90	1.41	0.0021	0.025	0.53	0.014	0.020	0.019
LPC 16:1/0:0	494.324	1.84	C24H48NO7P	5.7	0.0025	0.019	1.12	0.27	0.39	0.23	0.68	0.0014	0.013	1.35	1.65	7.0E-04	0.010	0.81	0.045	0.074	0.066
LPC 18:0/0:0	524.371	3.32	C26H54NO7P	4.1	0.40	0.40	1.39	0.24	0.35	0.50	1.16	0.43	0.47	0.43	1.20	0.67	0.77	0.40	0.37	0.45	0.32
LPC 18:1/0:0	580.362	2.7	C26H52NO7P	2.9	0.0050	0.051	1.11	0.11	0.24	0.37	0.86	0.031	0.11	0.70	1.29	0.0020	0.011	0.59	0.33	0.43	0.38
LPC 18:2/0:0	520.339	2.1	C26H50NO7P	3.5	0.39	0.39	1.10	0.56	0.65	0.33	0.87	0.33	0.36	0.66	1.27	0.21	0.32	0.47	0.19	0.23	0.21
LPC 20:3/0:0	546.355	2.57	C28H52NO7P	6.2	2.2E-04	0.0045	1.21	0.11	0.24	0.31	0.59	0.0029	0.018	1.65	2.04	2.5E-04	0.0064	1.07	0.0079	0.0160	0.0132
PC 14:1/20:2	756.553	6.79	C42H78NO8P	3.8	0.049	0.067	1.03	0.84	0.91	0.23	0.73	0.017	0.032	1.01	1.42	0.049	0.12	0.47	0.010	0.015	0.014
PC 16:0/18:1	818.591	9.24	C42H82NO8P	3.1	0.0090	0.072	1.02	0.81	0.92	0.24	0.80	0.0092	0.052	0.81	1.27	0.010	0.025	0.48	0.15	0.19	0.18
PC 16:0/20:3	842.591	8.35	C44H82NO8P	5.1	0.15	0.38	0.91	0.40	0.64	0.46	0.77	0.020	0.077	0.86	1.19	0.17	0.22	0.32	0.058	0.069	0.076
PC 16:0/22:4	810.601	8.28	C46H84NO8P	2.9	0.062	0.076	1.10	0.52	0.61	0.28	0.77	0.026	0.045	0.77	1.43	0.078	0.16	0.46	0.030	0.043	0.039
PC 16:0/16:1	732.553	7.14	C40H78NO8P	1.6	0.010	0.044	1.06	0.67	0.74	0.22	0.73	0.0099	0.025	1.13	1.45	0.020	0.070	0.56	0.10	0.15	0.14
PC 16:0/18:1	760.585	8.86	C42H82NO8P	1.0	0.0069	0.035	1.09	0.68	0.76	0.21	0.80	0.0043	0.020	0.79	1.37	0.012	0.062	0.44	1.01	1.38	1.27
PC 16:0/20:3	784.585	8.55	C44H82NO8P	1.9	1.7E-04	0.0045	1.15	0.26	0.37	0.29	0.44	0.0011	0.013	2.46	2.64	2.5E-04	0.0064	1.38	0.029	0.077	0.067
PC 16:0/22:4	810.601	8.66	C46H84NO8P	2.8	0.0019	0.018	0.85	0.069	0.20	0.30	0.71	2.5E-04	0.011	1.02	1.20	0.039	0.10	0.25	0.046	0.055	0.065
PC 18:0/20:3	870.622	11.29	C46H86NO8P	2.8	1.0E-04	0.0016	1.03	0.92	1.00	0.20	0.42	2.2E-05	0.0012	1.73	2.44	1.2E-04	0.0016	1.19	0.004	0.0108	0.011
PC 18:0/18:1	788.616	11.23	C44H86NO8P	1.9	0.026	0.058	0.99	0.90	0.94	0.19	0.80	0.0061	0.021	0.67	1.23	0.10	0.19	0.28	0.16	0.20	0.20
PC 18:0/20:5	808.585	8.06	C46H82NO8P	2.0	3.0E-04	0.0051	0.81	0.13	0.26	0.33	0.54	7.1E-04	0.013	1.81	1.49	4.0E-04	0.0083	0.57	0.049	0.073	0.090
PC 18:1/18:2	784.585	8.04	C44H82NO8P	1.9	0.039	0.061	0.91	0.54	0.64	0.32	0.70	0.0057	0.021	0.99	1.30	0.15	0.26	0.32	0.38	0.49	0.54
PC 18:2/22:2	838.631	11.06	C48H88NO8P	5.7	0.0013	0.015	0.74	7.8E-04	0.040	0.47	0.79	0.0032	0.018	0.74	0.95	0.45	0.57	0.12	0.018	0.017	0.023
PC 22:2/16:1	812.616	10.96	C46H86NO8P	4.2	1.5E-04	0.0045	1.15	0.34	0.45	0.27	0.47	1.8E-04	0.0033	2.18	2.45	2.0E-04	0.0064	1.25	0.016	0.040	0.034
PC 18:0/20:2	814.633	11.43	C46H88NO8P	3.9	0.013	0.047	0.89	0.22	0.36	0.25	0.77	0.0035	0.018	0.78	1.16	0.12	0.23	0.21	0.025	0.029	0.033
PC 22:4/18:1	836.616	10.25	C48H86NO8P	2.7	0.0024	0.019	0.82	0.092	0.21	0.30	0.59	1.9E-04	0.0033	1.61	1.39	0.0030	0.028	0.51	0.028	0.038	0.047
PC 22:0/24:1	928.768	15	C54H106NO8P	13.0	0.073	0.088	1.31	0.015	0.14	0.41	1.16	0.18	0.21	0.41	1.13	0.31	0.45	0.39	0.010	0.011	0.009
PC 16:0/7:0;CHO	622.407	3.26	C31H60NO9P	8.2	0.10	0.11	1.68	0.030	0.17	1.58	1.16	0.48	0.51	0.92	1.45	0.079	0.16	0.75	0.0026	0.0037	0.0022
PC 16:0/18:2;OH	774.564	4.59	C42H80NO9P	3.9	0.086	0.101	1.82	0.032	0.18	1.56	1.89	0.046	0.087	1.92	0.96	0.89	0.92	1.65	0.012	0.012	0.006
PC 16:0/20:4;5OH	798.563	4.7	C44H80NO9P	3.3	0.11	0.12	1.63	0.042	0.21	1.42	1.71	0.039	0.093	1.77	0.95	0.82	0.88	1.61	0.019	0.018	0.011
PC 18:0/18:2;OH	802.595	5.4	C44H84NO9P	6.5	0.058	0.073	1.87	0.022	0.15	1.52	1.72	0.043	0.11	1.87	1.08	0.76	0.83	0.64	0.004	0.005	0.003
PC 18:0/20:4;12OH	826.595	5.99	C46H84NO9P	12.6	0.13	0.14	1.03	0.91	0.99	1.51	1.46	0.023	0.042	1.52	0.71	0.29	0.41	1.39	0.011	0.008	0.008
Lyso-PAF C-16	482.361	2.9	C24H52NO6P	3.8	0.034	0.061	1.28	0.14	0.27	0.47	0.96	0.14	0.17	0.48	1.33	0.019	0.070	0.55	0.019	0.026	0.020
Glycerophosphoserines																					
LPS O-20:0;O	636.349	2.53	C26H54NO9P	1.5	0.11	0.30	1.13	0.34	0.58	0.31	0.91	0.18	0.26	0.59	1.25	0.046	0.079	0.48	0.43	0.53	0.47
PS 20:0/22:6	864.57	8.86	C48H82NO10P	18.7	0.016	0.049	1.09	0.34	0.47	0.16	0.83	0.029	0.049	0.60	1.32	0.011	0.062	0.41	0.076	0.100	0.091
PS 22:0/16:0	900.594	9.24	C44H86NO10P	1.8	0.016	0.102	1.03	0.76	0.91	0.24	0.84	0.0092	0.052	0.71	1.22	0.023	0.044	0.44	0.13	0.15	0.15
PS 22:0/20:0	893.7	13.55	C48H94NO10P	6.5	0.019	0.053	1.26	0.18	0.32	0.39	1.63	0.0049	0.019	1.30	0.77	0.15	0.26	0.57	1.65	1.27	1.01

Glycerophosphoglycerols																					
PG O-18:0/18:0	763.588	11.83	C42H85O9P	3.5	0.20	0.45	1.41	0.22	0.44	1.12	1.90	0.035	0.11	1.45	0.74	0.37	0.41	0.46	0.0167	0.012	0.009

							LGA vs AGA				SGA vs AGA				LGA vs SGA				*Semi-quantification in ppm*		
**Name**	***m/z***	**RT (min)**	**Formula**	**CV QC**	**p kruskal**	**p kruskal BH**	**FC**	***p* value**	***p*BH**	**VIP**	**FC**	***p* value**	***p*BH**	**VIP**	**FC**	***p* value**	***p*BH**	**VIP**	**ppm SGA (0.12ppm)**	**ppm LGA (0.12ppm)**	**ppm AGA (0.12ppm)**
**Organooxygen compounds**																					
Carbohydrates and carbohydrate conjugates																					
2-O-[alpha-D-Glucopyranosyl-(1->6)-alpha-D-glucopyranosyl]-D-glycerate	449.131	0.68	C15H26O14	2.1	0.056	0.23	1.01	0.72	0.94	0.14	0.75	0.038	0.11	0.71	1.35	0.053	0.084	0.50	0.36	0.48	0.48
4-O-Methyl-a-D-glucosyl-(1->2)-b-D-xylosyl-(1->4)-D-xylose	491.142	0.67	C17H28O15	2.9	0.0081	0.066	1.59	0.0073	0.043	0.92	1.00	1.00	1.00	0.31	1.59	0.0031	0.016	0.71	0.35	0.56	0.35
a-L-Fucopyranosyl-(1->2)-b-D-galactopyranosyl-(1->2)-D-xylose	477.162	0.67	C17H30O14	2.0	0.0073	0.063	2.24	0.0015	0.037	1.25	1.48	0.037	0.11	0.93	1.51	0.078	0.10	0.66	0.38	0.57	0.25
**Prenol lipids**																					
Quinone and hydroquinone lipids																					
Vitamin K1 epoxide	501.311	0.92	C31H46O3	3.9	0.033	0.17	0.44	0.58	0.84	0.56	18.56	0.11	0.19	2.24	0.02	0.010	0.025	1.89	0.0056	0.00013	0.00030
**Purines and purine derivatives**																					
Caffeine	195.088	0.78	C8H10N4O2	8.6	0.083	0.098	0.43	0.077	0.20	1.42	0.37	0.018	0.035	4.49	1.15	0.22	0.34	1.41	0.00016	0.00018	0.00043
**Sphingolipids**																					
Phosphosphingolipids																					
SM 18:1;O2/21:0	811.604	8.66	C44H89N2O6P	3.0	0.0026	0.019	0.87	0.11	0.24	0.27	0.73	3.0E-04	0.011	0.95	1.20	0.042	0.11	0.25	0.019	0.023	0.026
SM 16:0;O2/22:0	761.653	11.51	C43H89N2O6P	4.8	0.026	0.058	1.15	0.060	0.19	0.19	0.93	0.13	0.16	0.27	1.24	0.0099	0.055	0.30	0.014	0.017	0.015
SM 18:1;O2/20:0	819.659	11.64	C43H89N2O6P	5.3	0.018	0.11	1.11	0.21	0.42	0.37	0.85	0.016	0.084	0.69	1.31	0.0059	0.020	0.60	0.0020	0.0026	0.0023
SM 18:0;O2/22:0	789.686	11.94	C45H93N2O6P	6.2	0.0033	0.021	1.07	0.26	0.37	0.11	0.84	0.011	0.026	0.52	1.27	0.0029	0.028	0.36	0.013	0.017	0.016
SM 18:1;O2/22:0	787.668	11.54	C45H91N2O6P	7.0	0.0070	0.035	1.36	0.018	0.14	0.43	0.92	0.29	0.32	0.31	1.47	0.0049	0.046	0.52	0.0068	0.0100	0.0074
SM 18:1;O2/24:0	815.7	11.93	C47H95N2O6P	2.2	0.014	0.047	1.24	0.015	0.14	0.31	0.97	0.61	0.64	0.19	1.28	0.0070	0.055	0.35	0.049	0.063	0.050
SM 18:0;O2/26:0	845.741	13.17	C49H101N2O6P	12.0	0.037	0.061	1.40	0.098	0.22	0.49	1.54	0.0082	0.020	1.29	0.90	0.31	0.44	0.47	0.020	0.018	0.013
Ceramides																					
Cer 18:0;O3/26:0;O	692.656	12.56	C44H89NO5	4.6	0.046	0.22	0.81	0.090	0.21	0.72	1.11	0.21	0.28	0.45	0.73	0.010	0.024	0.63	0.00018	0.00013	0.00016
**Sterol lipids**																					
Bile acids and derivatives																					
ST 24:3;O4	447.275	0.96	C24H36O4	8.1	0.16	0.40	0.64	0.050	0.14	0.73	0.72	0.12	0.20	0.74	0.89	0.53	0.54	0.24	0.0019	0.0017	0.0027
Neurosteroid																					
Pregnanolone sulfate	397.205	0.79	C21H34O5S	4.0	0.026	0.15	1.46	0.0051	0.048	0.62	1.20	0.15	0.23	0.55	1.21	0.16	0.20	0.30	0.0042	0.0050	0.0035
Steroid conjugates																					
Estriol 3-sulfate 16-glucuronide	543.155	0.66	C24H32O12S	5.3	0.049	0.23	2.01	0.051	0.14	0.98	1.08	0.68	0.73	0.54	1.86	0.060	0.084	0.65	0.0020	0.0038	0.0019
eurysterol B sulfonic acid	493.263	0.9	C27H44O7S	3.4	0.12	0.33	1.35	0.036	0.11	0.58	1.03	0.83	0.87	0.42	1.30	0.076	0.11	0.52	0.0020	0.0038	0.0019
17-beta-estradiol 3-sulfate-17-(beta-D-glucuronide)	509.153	0.67	C24H32O11S	8.3	0.021	0.13	1.90	0.0070	0.043	1.13	0.97	0.85	0.88	0.29	1.95	0.0053	0.020	0.83	0.0046	0.0060	0.0045
Sterols																					
32-oxolanosterol	499.379	6.06	C30H48O2	3.9	0.053	0.23	3.74	0.093	0.24	1.40	2.76	0.016	0.073	1.85	1.35	0.75	0.75	0.36	0.15	0.20	0.054
22:0-Glc-Cholesterol	871.742	13.25	C55H98O7	2.4	0.030	0.059	1.10	0.67	0.75	0.21	1.45	0.0077	0.020	1.03	0.76	0.078	0.16	0.49	0.0092	0.007	0.0063
22:2-Glc-Sitosterol	912.765	13.19	C57H98O7	4.1	0.035	0.061	0.99	0.95	0.98	0.18	1.49	0.015	0.031	1.08	0.67	0.016	0.064	0.66	0.0219	0.0146	0.0147
CE 16:1	640.602	14.57	C43H74O2	3.6	0.020	0.054	0.93	0.68	0.75	0.42	0.61	0.0014	0.014	1.76	1.53	0.079	0.16	0.60	0.0054	0.0082	0.0088
CE 18:0	1306.24	16.27	C45H80O2	5.4	0.27	0.28	0.73	0.14	0.27	0.93	0.71	0.050	0.082	0.86	1.03	0.90	0.92	0.43	0.0055	0.0057	0.0078
CE 18:3	664.602	14.11	C45H74O2	22.0	0.013	0.047	0.89	0.18	0.32	0.57	0.61	7.9E-04	0.013	1.61	1.46	0.40	0.55	0.53	0.0012	0.0017	0.0020
CE 20:3	713.563	14.69	C47H78O2	2.9	0.0066	0.035	0.89	0.19	0.33	0.24	0.77	9.1E-04	0.013	0.73	1.16	0.079	0.16	0.22	0.0061	0.0070	0.0079
DiMe(9,3) Cholesterol ester	663.571	12.71	C45H74O3	11.1	0.048	0.067	2.87	0.042	0.15	1.82	2.16	0.049	0.093	2.05	1.33	0.93	0.96	1.59	0.0328	0.0435	0.0152
Cholesteryl 11-hydroperoxy-eicosatetraenoate	743.538	12.41	C47H76O4	3.2	1.0E-04	0.0045	0.52	0.049	0.18	1.95	2.46	0.038	0.026	2.72	0.21	3.7E-05	0.0038	1.97	0.27	0.057	0.11
Cholesteryl-6-O-myristoyl-alpha-D-glucoside	739.589	12.57	C47H82O7	3.0	0.012	0.092	1.32	0.97	1.00	1.53	3.37	0.0033	0.045	1.69	0.39	0.035	0.064	1.36	0.0037	0.0015	0.0011
Sitostanyl-18:0	721.625	12.71	C47H86O2	21.4	0.099	0.11	3.82	0.034	0.20	1.96	2.87	0.031	0.11	2.21	1.33	0.98	0.99	1.77	0.16	0.21	0.055
ST 29:2;O3	445.368	7.76	C29H48O3	19.1	0.23	0.25	0.73	0.34	0.33	2.07	1.11	0.67	0.69	0.50	0.66	0.13	0.23	1.86	0.0046	0.0031	0.0042
(±)9-HODE cholesteryl ester	647.575	12.47	C45H76O3	10.1	0.14	0.15	1.15	0.24	0.35	0.62	1.63	0.048	0.073	1.64	0.70	0.13	0.22	0.85	0.0035	0.0024	0.0021
Secosteroids																					
1alpha,25-dihydroxy-16,17,23,23,24,24-hexadehydrovitamin D3	445.255	3.63	C27H38O3	5.6	0.053	0.23	0.91	0.81	0.97	0.08	1.32	0.11	0.19	0.85	0.69	0.011	0.025	0.59	0.10	0.070	0.077
3-Deoxyvitamin D3	369.351	12.52	C27H44	4.9	0.047	0.067	1.16	0.49	0.59	1.58	1.88	0.011	0.030	2.02	0.62	0.048	0.12	1.71	0.38	0.24	0.20

CV, coefficient of variation of lipids in QC samples; p Kruskal, global p value obtained by Kruskal-Wallis test; p Kruskal BH, global p value obtained by Benjamini-Hochberg correction test; p value: obtained by Mann-Whitney U tests. pBH, Benjamini-Hochberg correction test p value; FC, fold change in the specified comparison; VIP, Variable importance in projection values higher than 1 were considered as significant.
